# First identification of porcine parvovirus 6 in Poland

**DOI:** 10.1007/s11262-016-1386-y

**Published:** 2016-09-02

**Authors:** Jin Cui, Jinghui Fan, Priscilla F. Gerber, Kinga Biernacka, Tomasz Stadejek, Chao-Ting Xiao, Tanja Opriessnig

**Affiliations:** 1 The Roslin Institute and The Royal (Dick) School of Veterinary Studies, University of Edinburgh, Midlothian, UK; 2 College of Veterinary Medicine, Agricultural University of Hebei, Baoding, People’s Republic of China; 3 Department of Pathology and Veterinary Diagnostics, Faculty of Veterinary Medicine, Warsaw University of Life Sciences, Warsaw, Poland; 4 Department of Veterinary Diagnostic and Production Animal Medicine, College of Veterinary Medicine, Iowa State University, Ames, IA USA; 5 College of Biology, Hunan University, Changsha, People’s Republic of China

**Keywords:** Porcine parvovirus 6, Phylogenetic analysis, Poland, Pigs

## Abstract

Porcine parvovirus type 1 is a major causative agent of swine reproductive failure. During the past decade, several new parvoviruses have been discovered in pigs. Porcine parvovirus type 6 (PPV6), recently identified, has been reported in pigs in China and in the USA while the PPV6 status in the European pig population remains undetermined. In the present study, PPV6 DNA was identified in serum samples collected from domestic pigs in Poland. In investigated herds, the prevalence of PPV6 was 14.9 % (15/101 samples). Sequencing was conducted, and 11 nearly complete PPV6 genomes were obtained. Phylogenetic analysis indicated that PPV6 sequences cluster into four distinct groups, and the Polish PPV6 strains from three individual farms were present in three of these four groups. In addition, the Polish PPV6 strain P15-1 was identified as a putative recombination of an ORF1 from US stains and an ORF2 from Chinese strains. This is the first identification of PPV6 in Europe, and this finding will encourage future epidemiological studies on parvoviruses in European pigs.

**Electronic supplementary material:**

The online version of this article (doi:10.1007/s11262-016-1386-y) contains supplementary material, which is available to authorized users.

## Introduction

Parvoviruses are small, non-enveloped DNA viruses with a single-stranded linear genome of approximately 4.0–6.3 kb in size containing two to three main open reading frames (ORFs) [1, 2]. The family *Parvoviridae* consists of two subfamilies: the *Parvovirinae* infecting vertebrates and the *Densovirinae* infecting arthropods. The subfamily *Parvovirinae* can be further divided into eight genera [3] of which *Protoparvovirus*, *Bocaparvovirus*, *Copiparvovirus*, and *Tetraparvovirus* contain viruses that infect pigs.

Porcine parvovirus 1 (PPV1) is a well-known pathogen in pigs and frequently associated with reproductive failure in breeding herds, resulting in widespread breeding herd vaccination in an effort to control this virus [4]. PPV1 was first isolated in Germany in 1965 as a contaminant of cell cultures used for classical swine fever virus propagation [5]. During the past decade, several new species of parvoviruses have been identified in pigs commonly known as PPV2 through PPV6. Unlike PPV1 which belongs to the genus *Protoparvovirus*, the emerging PPV species belong to the genera *Tetraparvovirus* (PPV2, PPV3) and *Copiparvovirus* (PPV4, PPV5, and PPV6) [3].

PPV6 shares only 20.5–42.6 % nucleotide similarity with other members of the subfamily *Parvovirinae* and is most closely related to PPV4 [6]. PPV6 was first identified in aborted pig fetuses in China in 2014 and subsequently was also reported in the USA [6, 7]. The distribution of PPV6 in the global pig population and possible clinical signs associated with PPV6 infection remain to be determined. In this study, PPV6 DNA was identified in serum samples collected from domestic Polish pigs and further characterized by genomic sequencing. Eleven nearly complete PPV6 sequences were obtained. To our knowledge, this study is the first to describe PPV6 in European pigs.

## Materials and methods

Serum samples (*n* = 101) were collected from 2- to 18-week-old commercial cross-bred pigs located on three commercial Polish pig farms designated as U, K, and P. The herds ranged in size from 600 to 1000 pigs. Total nucleic acids were extracted from serum samples using the MagMAX™ Viral RNA Extraction Kit (Thermo Scientific) on an automated extraction system (Thermo Scientific Kingfisher Flex) according to the manufacturer’s instructions. The extracted samples were stored at −20 °C until testing.

Initial detection of PPV6 DNA in the sample set was done by a real-time PCR developed based on the alignments of PPV6 available in the GenBank. A pair of primers (PPV6F3250:5′-GGCTTCATAATCCCTCCAAAACCT-3′, PPV6R3404:5′-GCTCATCTTCCTCTTGTTTCTCCTG-3′) targeting 154 bp in ORF2 and a specific TaqMan^®^ probe (PPV6prob: Cal Fluor Orange 560-5′-CCTCCTCCTCCTCCCTCTCCAATTCCT-3′-BHQ1) within the region of the detection primers were designed using Primer Express software (Version 3.0; Applied Biosystems). The PCRs were carried out in 96-well plates, with each reaction containing a total volume of 25 μl, 12.5 μl of the TaqMan^®^ Universal PCR Master Mix (Applied Biosystems^®^), 2.5 μl of the sample or standard DNA, 1 μl of 10 μM of each of the two primers, 0.5 μl of 10 μM probe, and 7.5 μl nuclease-free water. Amplification reactions were performed using the ABI 7500 Fast Real-Time PCR System (Applied Biosystems^®^) under universal conditions: 2 min at 50 °C, 10 min at 95 °C, 40 cycles of 15 s at 95 °C, and 1 min at 60 °C. Samples with no cycle threshold (*C*_T_) at 37 cycles were considered negative. The specificity of the probe was confirmed by BLAST analysis and using DNA samples positive for other DNA viruses including PPV1 through PPV5, porcine circoviruses 1 and 2, and Torque teno sus virus. The sensitivity of the PPV6 real-time PCR assay was determined by testing 10-fold serial dilutions of the PPV6 DNA standard in triplicate. The generated standard curve with a regression value of *R* ^2^ = 0.989 was linear and showed a reaction efficiency of 114.092. The detection limit was 50 genome copies per reaction with Ct values around 36 in all three replicates. Real-time positive samples were also tested by a conventional PCR assay as previously described [6]. To further characterize PPV6 DNA positive samples, a set of overlapping primers was designed to amplify the nearly complete PPV6 genome (Table S1). The amplifications were performed using the Q5 High-Fidelity PCR kit (NEB) in a 50 µl volume containing 25 µl of Q5 High-Fidelity 2× mix, 2 µl of a 10 µM forward primer, 2 µl of a 10 µM reverse primer, 2 µl of the extracted DNA, and 19 µl of ddH_2_O under the following thermocycler conditions: denaturation for 30 s at 98 °C followed by 30 cycles of 98 °C for 10 s, 55 °C for 30 s, and 72 °C for 60 s, and a final extension step at 72 °C for 10 min. The PCR products were purified by the PureLink™ Quick Gel Extraction and PCR Purification Combo Kit (Invitrogen, Life Technologies) according to the manufacturer’s instructions and were sequenced at the Edinburgh Genomics facility, University of Edinburgh, Edinburgh, United Kingdom. The sequences were assembled by the SeqMan program of Lasergene 7.0 software (DNASTAR Inc., Madison, Wisconsin, USA) and compared with other available PPV6 sequences using the BLAST web-based program (http://www.ncbi.nlm.nih.gov/BLAST). PPV6 sequences were then aligned with available reference sequences downloaded from GenBank by Clustal Omega (http://www.ebi.ac.uk/). Phylogenetic trees were inferred by maximum-likelihood (ML) method (amino acid sequences) or neighbor-joining method (nucleotide sequences) implemented in MEGA v7.0 [8]. Support for individual nodes was determined by 1000 bootstrap replicates.

## Results and discussion

**Fig. 1:**
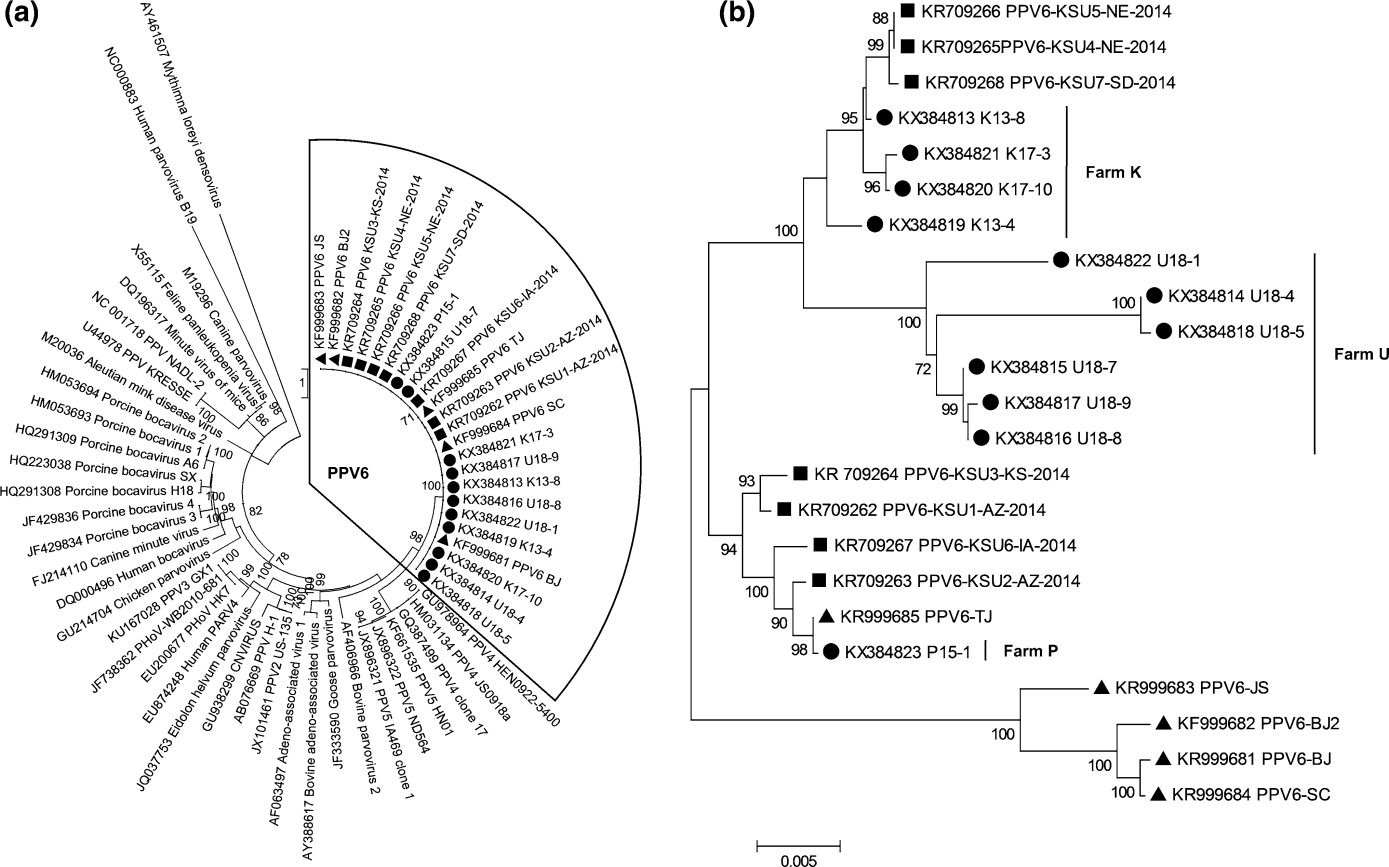
Phylogenetic analysis of porcine parvovirus 6 (PPV6) sequences obtained in Poland (*black circles*). **a** Phylogenetic relationship of PPV6 within subfamily *Parvovirinae.* The phylogenetic tree was inferred from amino acid sequences of the non-structural protein (ORF1, NS1) by the ML method with the LG+F+I model. **b** The phylogenetic tree of the nucleotide sequences of the capsid protein (ORF2, VP1) of PPV6 was constructed by neighbor-joining method based on Kimura 2-parameter model. Bootstrap values (1000 replicates) <70 % are not shown. The Polish strains are represented by *black circles*, Chinese strains are indicated by *black triangles* and US strains are represented by *black squares*. *Scale bars* indicate the number of substitutions per site

In the present study, among the 101 serum samples from three different Polish farms, PPV6 DNA was detected in 14.9 % (15/101) of the samples. The individual PPV6 farm prevalence rate ranged from 3.8 % for farm P to 22.2 % for farm K (Table S2). Eleven of the PCR positive samples were sequenced (Table S2), and the nucleotide sequences of these nearly complete PPV6 genomes were deposited into GenBank under the accession numbers KX384822 (U18-1), KX384814 (U18-4), KX384818 (U18-5), KX384815 (U18-7), KX384816 (U18-8), KX384817 (U18-9), KX384819 (K13-4), KX384813 (K13-8), KX384821 (K17-3), KX384820 (K17-10), and KX384823 (P15-1). The complete coding regions (excluding 5′ and 3′ UTR) of the Polish PPV6 strains were 5566 nucleotides (nt) in length and contained two major open reading frames (ORFs). ORF1 (1989 nt) encoded the NS1 protein with 663 amino acids (aa) and ORF2 (3570 nt) encoded the VP1 protein with 1190 aa. Based on amino acid sequences of the NS1 protein which is commonly used to differentiate members of the subfamily *Parvovirinae*, all PPV6 strains identified in Poland were closely related to PPV6 reference strains and located in the genus *Copiparvovirus* together with PPV4 and PPV5 (Fig. 1a) as described previously [6, 7].

The complete coding regions of the identified PPV6 strains shared 98.0–99.8 % nucleotide sequence identity with US PPV6 strains and 96.4–98.9 % nucleotide sequence identity with Chinese PPV6 strains. Sequences isolated from the same farm showed a high nucleotide identity (98.6–99.9 %) to each other. The motifs of the calcium binding loop (YXGXR) and the catalytic residues (HDXXY) of PLA2 were found to be conserved among PPV6 strains. Compared with NS1, VP1 resembles the variable protein in the PPV6 genome. The VP1 sequences of the investigated Polish PPV6 strains showed 94.3–100 % nucleotide identity (95.0–100 % amino acid identity) with reference sequences. A previous study indicated that there may be three conserved regions in the PPV6 VP1 protein [7]. However, compared with reference PPV6 sequences identified in China or in the USA, the first two regions were not conserved (Table 1). In the first region (aa 164-256), four amino acid mutations were observed, three amino acid changes were present in Farm U PPV6 strains (U18-1, U18-4, U18-5, U18-8 and U18-9), and the other one was present in a Farm K PPV6 strain (K17-3). Furthermore, Farm U strains contained a total of 28 mutations in the second region (aa 414-787). Phylogenetic analysis of the VP1 gene showed that PPV6 strains clustered in four distinct groups (Fig. 1b). While the PPV6 sequences from Farm K were closely related to the three US strains, Farm U sequences, which shared most mutations in the putative conserved regions, formed a distinct cluster. Interestingly, strain P15-1 from Farm P shared 99.9 % homology (one nucleotide difference) with the US strain KSU7-SD-2014 (GenBank accession no. KR709268) in the ORF1 region. In contrast, in the ORF2 region, P15-1 showed 99.9 % similarity (one nucleotide difference) with the Chinese strain TJ (GenBank accession no. KF999685). Moreover, phylogenetic analysis showed that P15-1 clustered with different strains in NS1 and VP1 regions, indicating a possible recombination event for this strain.

**Table 1:** Amino acid substitutions in putative conserved regions of the Polish porcine parvovirus type 6 (PPV6) VP1

Amino acid position	KSU7-SD-2014 KR709268	TJ KF999685	K17-3 KX384821	U18-1 KX384822	U18-4 KX384814	U18-5 KX384818	U18-7 KX384815	U18-8 KX384816	U18-9 KX384817
167 173 216 217 518 543 583 586 610 629 645 661 664 667 676 696 697 706 713 728 730 732 734 739 740 741 742 743 744 747 752 773	Q I W R D R T E G G I Y S H E W Q F V E Q T Q A T I A I F K P L	– – – – – – – – – – – – – – – – – – – – – – – – – – – – – – – –	– – – G – – – – – – – – – – – – – – – – – – – – – – – – – – – –	H – – – – – I D – – – – – – – – – – – – – – – – – – – – – – T –	– V – - H T I D R R V H F P K C L L G K L K L V R H C F L I – –	– V – – H T I D R R V H F P K C L L G K L K L V R H C F L I – –	– – – – – – I D – – – – – – – - – – – – – – – – – – – – – – – –	– – G – – – I D – – – – – – – – – – – – – – – – – – – – – – – –	– – G – – – I D – – – – – – – – – – – – – – – – – – – – – T H

KSU7-SD-2014 (KR709268) represents a US PPV6 reference strain and TJ (KF999685) represents a Chinese reference strains. K17-3 (KX384821), U18-1 (KX384822), U18-4 (KX384814), U18-5 (KX384818), U18-7 (KX384815), U18-8 (KX384816), and U18-9 (KX384817) are Polish PPV6 strains identified in this study. Dashes indicate conserved amino acid positions compared to strain SKU7-SD-2014

## Conclusions

Our findings demonstrate that PPV6 circulates in swine herds in Poland. This study represents the first identification of PPV6 in European pigs, indicating that PPV6 is not restricted to Asia and North America. Phylogenetic analysis suggests that the PPV6 P15-1 strain might be a chimeric virus containing an ORF1 gene from the USA and an ORF2 gene from China. Further studies are ongoing to investigate the epidemiology and evolution of parvovirus in European pigs. To date, the exact relatedness of pathogenesis and occurrence of novel species of PPV strains in pigs remains unknown.

## Electronic supplementary material

Below is the link to the electronic supplementary material.

Supplementary material 1 (DOCX 13 kb)

## References

[1] Tijssen P, Agbandje-McKenna M, Almendral JM, Bergoin M, Flegel TW, Hedman K, Kleinschmidt J, Li Y, Pintel DJ, Tattersall P, King AMQ, Adams MJ, Carstens EB, Lefkowitz EJ (2011). The family Parvoviridae. Virus taxonomy—Ninth Report of the International Committee on Taxonomy of Viruses.

[2] Xiao CT, Giménez-Lirola LG, Jiang YH, Halbur PG, Opriessnig T (2013). PLoS One.

[3] Streck AF, Canal CW, Truyen U (2015). Infect. Genet. Evol..

[4] Hueffer K, Parrish CR (2003). Curr. Top. Microbiol. Immunol..

[5] Mayr A, Mahnel H (1964). Zentralbl. Bakteriol. Orig..

[6] Ni J, Qiao C, Han X, Han T, Kang W, Zi Z, Cao Z, Zhai X, Cai X (2014). Virol. J..

[7] Schirtzinger EE, Suddith AW, Hause BM, Hesse RA (2015). Virol. J..

[8] Tamura K, Peterson D, Peterson N, Stecher G, Nei M, Kumar S (2011). Mol. Biol. Evol..

