# Supplementary material for: First identification of porcine parvovirus 6 in Poland
Source: Virus Genes. 2016 Sep 2;53(1):100–4. doi: 10.1007/s11262-016-1386-y (PMC5306181; doi:10.1007/s11262-016-1386-y)
Supplement: Supplementary file 1 — Supplementary material 1 (DOCX 13 kb) [file 11262_2016_1386_MOESM1_ESM.docx]

**Table S1**

Porcine parvovirus type 6 (PPV6) sequencing primers.

| **Primers** | **Sequences (5’-3’)** | **Position** | **Product size (bp)** |
| --- | --- | --- | --- |
| PPV6-1-F | CGAAAGCCTCTGTATTATGTCT | 203 | 1109 |
| PPV6-1-R | TTGCGTTATTCCAGTTAACAC | 1291 |  |
| PPV6-2-F | TGTTTGGACCTGCTACAACCG | 1219 | 1286 |
| PPV6-2-R | TTAAAACCCTGAACACCGGAA | 2484 |  |
| PPV6-3-F | CTCTCAGTCAATCCGACGTT | 2113 | 759 |
| PPV6-3-R | ATTCTGACGCCAATTATCAGC | 2851 |  |
| PPV6-4-F | CAGATCCCCTATATGCTAGCC | 2821 | 807 |
| PPV6-4-R | ATCTCGCTGAGCATTATCCAA | 3607 |  |
| PPV6-5-F | TCTTAAACATGGAGACTGGC | 3567 | 1107 |
| PPV6-5-R | CCTCGAATAAGAGTGGCATC | 4654 |  |
| PPV6-6-F | CAGTCAGCCACCATTGCTA | 4423 | 916 |
| PPV6-6-R | CTTTTGGAATGGTAACATCCCC | 5317 |  |
| PPV6-7-F | AGGTTAGCCTATCAACGTC | 5236 | 602 |
| PPV6-7-R | TCAACCAGAATGCCTGACAC | 5818 |  |

**Table S2**

Farm origin of the obtained Porcine parvovirus type 6 (PPV6) sequences, GenBank accession numbers, and the PPV6 prevalence rates on those farms.

| **Farm ID** | **Prevalence rate** | **Strains** | **GenBank No.** |
| --- | --- | --- | --- |
| **U** | 15.4% (6/39) | U18-1 | KX384822 |
|  |  | U18-4 | KX384814 |
|  |  | U18-5 | KX384818 |
|  |  | U18-7 | KX384815 |
|  |  | U18-8 | KX384816 |
|  |  | U18-9 | KX384817 |
| **K** | 22.2% (8/36) | K13-4 | KX384819 |
|  |  | K13-8 | KX384813 |
|  |  | K17-3 | KX384821 |
|  |  | K17-10 | KX384820 |
| **P** | 3.8% (1/26) | P15-1 | KX384823 |
